# A novel use of inertial sensors to measure the craniocervical flexion range of motion associated to the craniocervical flexion test: an observational study

**DOI:** 10.1186/s12984-020-00784-1

**Published:** 2020-11-19

**Authors:** Tomás Pérez-Fernández, Susan Armijo-Olivo, Sonia Liébana, Pablo José de la Torre Ortíz, Josué Fernández-Carnero, Rafael Raya, Aitor Martín-Pintado-Zugasti

**Affiliations:** 1 Departamento de Fisioterapia, Facultad de Medicina, Universidad San Pablo-CEU, CEU Universities, Madrid, Spain; 2 Faculty of Business and Social Sciences, University of Applied Sciences, Caprivistr, 30A, 49076 Osnabrück, Germany; 3 Department of Physical Therapy, Faculty of Rehabilitation Medicine, University of Alberta, 3-48 Corbett Hall, Edmonton, AB Canada; 4 Department of Physical Therapy, Occupational Therapy, Rehabilitation and Physical Medicine, Rey Juan Carlos University, Madrid, Spain; 5 La Paz Hospital Institute for Health Research, IdiPAZ, Madrid, Spain; 6 Grupo Multidisciplinar de Investigación y Tratamiento del Dolor, Grupo de Excelencia Investigadora URJC-Banco de Santander, Madrid, Spain; 7 Departmento de Ingeniería de Sistemas de Información, Universidad San Pablo-CEU, CEU Universities, Madrid, Spain; 8 Werium Solutions, Arganda del Rey, 28500 Madrid, Spain

**Keywords:** Movement disorders, Neck muscles, Neck pain, Temporomandibular joint disorders, Reproducibility, Exercise, Headache

## Abstract

**Background:**

The craniocervical flexion test (CCFT) is recommended when examining patients with neck pain related conditions and as a deep cervical retraining exercise option. During the execution of the CCFT the examiner should visually assess that the amount of craniocervical flexion range of motion (ROM) progressively increases. However, this task is very subjective. The use of inertial wearable sensors may be a user-friendly option to measure and objectively monitor the ROM. The objectives of our study were (1) to measure craniocervical flexion range of motion (ROM) associated with each stage of the CCFT using a wearable inertial sensor and to determine the reliability of the measurements and (2) to determine craniocervical flexion ROM targets associated with each stage of the CCFT to standardize their use for assessment and training of the deep cervical flexor (DCF) muscles.

**Methods:**

Adults from a university community able to successfully perform the CCFT participated in this study. Two independent examiners evaluated the CCFT in two separate sessions. During the CCFT, a small wireless inertial sensor was adhered to the centre of the forehead to provide real-time monitoring and to record craniocervical flexion ROM. The intra- and inter-rater reliability of the assessment of craniocervical ROM was calculated. This study was approved by the Research Ethics Committee of CEU San Pablo University (236/17/08).

**Results:**

Fifty-six participants (18 males, 23 females; mean [SD] age, 21.8 [3.45] years) were included in the study and successfully completed the study protocol. All interclass correlation coefficient (ICC) values indicated good or excellent reliability of the assessment of craniocervical ROM using a wearable inertial sensor. There was high variability between subjects on the amount of craniocervical ROM necessary to achieve each stage of the CCFT.

**Conclusions:**

The use of inertial sensors is a reliable method to measure the craniocervical flexion ROM associated with the CCFT. The great variability in the ROM limits the possibility to standardize a set of targets of craniocervical flexion ROM equivalent to each of the pressure targets of the pressure biofeedback unit.

## Background

Neck pain is one of the main causes of disability throughout the world, being the fourth leading cause of years lost to disability globally [1]. The annual prevalence of neck pain ranges between 15 and 50%, and it has been estimated that its lifetime prevalence is close to half of the world’s population, who will suffer at least one neck pain episode over the course of their lifetime [2, 3].

Neck pain has been related with multiple impairments of the cervical sensorimotor system, such as reduced muscle strength and endurance of the cervical muscles [4–6], altered proprioception [7, 8], impaired kinematics [9–11] or changes in muscle morphology [12].

Deep cervical flexor (DCF), longus colli and capitis muscles provide cervical segmental support and stability. It has been reported that patients with neck pain, cervicogenic headache, temporomandibular disorders (TMDs) and craniofacial pain show alterations of their function and structure [12–16]. In addition, these muscles showed reduced electromyographic activity or delayed onset of activation [13–20]. Therefore, the performance of the DCF muscles is frequently considered in the evaluation of patients with neck pain and related conditions [21, 22], as well as in the prescription of specific therapeutic exercise programmes [23].

The craniocervical flexion test (CCFT) specifically assesses the function of the DCF muscles. This test involves controlled upper cervical flexion action in five incremental stages of the craniocervical range of motion (ROM) [24, 25]. An air-filled pressure sensor placed behind the neck serves as the feedback to monitor the subtle flattening of the cervical lordosis caused by the contraction of the longus colli muscle. A correct performance of the test implies the ability to achieve and maintain an isometric contraction at each of the pressure stages without any compensatory movement or excessive use of the superficial cervical flexors [24]. The construct validity of the CCFT has been verified through electromyography studies, showing how the five stages of the CCFT are associated with a progressive activation of the DCFs [13–15]. Patients with neck pain as well as other conditions associated with it have shown a lower performance of the CCFT as compared to asymptomatic individuals [13, 15, 26, 27].

International guidelines that help lead physical therapists with regard to the examination, diagnosis and treatment of neck pain include the CCFT in the expected exam findings when examining patients with neck pain and cervicogenic headache [28]. Moreover, international consensus experts suggested it should be used to evaluate myogenic and mixed TMDs and chronic orofacial pain [22].

The CCFT is also used as a retraining exercise option in patients with neck pain, TMDs or headaches. A recent systematic review has reported that the low-load CCFT exercise may effectively train the DCF muscles and alleviate chronic neck pain, being the primary exercise option for patients with deep craniocervical flexor impairments [29].

Previous research using digital imaging techniques confirmed that the amount of craniocervical flexion ROM progressively increases during the five consecutive stages of the CCFT, representing the increasing contractile demand of the DCF muscles [14, 15, 30, 31]. One of the components of the performance of the CCFT is to visually assess the quality and range of head rotation in the sagittal plane during the test to confirm that it proportionally increases with progressive stages of the test [15, 24]. Therefore, it is important that clinicians are able to detect this motion and possible aberrant strategies when conducting the CCFT.

The use of inertial wearable sensors may be a user-friendly option to measure and objectively monitor the ROM associated with each stage of the CCFT, while computer feedback could guide the performance of the test for both the patient and examiner.

Moreover, since the CCFT is performed in the supine position, the air-filled pressure pad needs to be compressed between the cervical lordosis of the patient and the bed. With regard to this, previous research has already suggested that training of the cervical muscles should include functional postures and tasks [32]. Therefore, a device that accomplishes these demands is needed. Thus, inertial sensors associated with computer feedback could provide an alternative option to test and retrain the DCF muscles in multiple positions or functional activities.

The objectives of the present study are:

1. To measure craniocervical flexion ROM associated with each stage of the CCFT using a wearable inertial sensor and to determine the reliability of the measurement.
2. To determine craniocervical flexion ROM targets associated with each stage of the CCFT using a wearable inertial sensor to standardize their use for assessment and training of the DCF muscles.

## Methods

### Design

This is a methodological study that looked at the reliability of the assessment of craniocervical flexion ROM associated with the CCFT using a wearable inertial sensor. The study follows a reproducibility protocol in three stages, including training, overall agreement, and study phase [33]. The training and overall agreement stages have already been completed and constituted the basis of the methods used on the present observational study. The present study addresses the study phase of the reproducibility protocol. In the training and overall agreement periods, the examiners discussed and agreed on the definition of the performance of the CCFT and the use of the inertial wearable sensors described in this section.

The study was approved by the Research Ethics Committee of Ceu San Pablo University (236/17/08). Participants provided informed written consent before being enrolled in the study and were able to withdraw their consent at any time during the study**,** in compliance with the WHO standards and the Declaration of Helsinki [34].

### Sample and selection

The participants to be investigated in this study were asymptomatic adults from a university community. Subjects were recruited through various channels and methods in a university campus, including advertisement, word of mouth and emailing.

Participants were eligible to be included in the study if they were between 18 and 30 years old with no known conditions affecting the craniocervical region in the past year and were able to successfully perform the five stages of the CCFT (the detailed procedure is described in the section on instrumentation and measures).

Subjects were excluded if they presented with any of the following criteria: (a) history of pain in the craniocervical region and/or shoulders at the time of the measurement or during the previous year, (b) history of surgery in the head or neck area, (c) previous diagnosis of TMDs, headaches or (d) any neurological deficits.

A sample of asymptomatic participants were chosen, since it was considered is the most appropriate sample to accomplish the objectives of the present study. The descriptive results of craniocervical flexion ROM associated with the CCFT could represent the normative values to provide a standardization that could posteriorly serve as a reference point for patients with pain conditions in future studies.

Once deemed eligible, subjects were invited to participate in the study and were requested to read and sign the informed consent prior to participation.

### Instrumentation and measures

Prior to testing, the demographic characteristics of the subjects, such as age, gender, weight, height and hours of physical activity/week, were recorded.

**Fig. 1:**
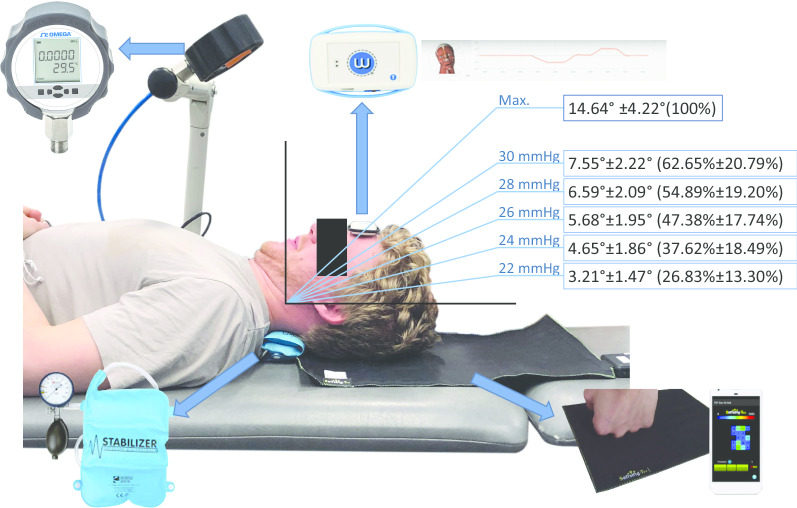
Experimental setup. Data show the mean ROM and percentage of the full range of craniocervical flexion in each stage of the CCFT

The CCFT was performed as described in the most recent clinical protocol by Jull et al. [24]. First, subjects were asked to perform the CCFT to assess whether they were able to successfully perform the five stages of the CCFT. If they were able to do it, they were included; thus, this procedure also served as a warm-up and a training for the performance of the CCFT before the real testing. Participants were placed in a relaxed supine position with the knees flexed, forearms resting on the abdomen and the neck in a neutral position with the face and the line bisecting the neck longitudinally being horizontal to the plinth. The examiner placed an uninflated pressure sensor (Pressure Biofeedback Unit, Stabilizer™ Chattanooga Group, Hixon, TN, USA) behind the neck of the patient and inflated it to a pressure of 20 mmHg. It was connected to a high accuracy digital pressure gauge (1/4ʺ NPT M, 5 PSI; DPG210; Omega Engineering Limited, UK) through a flexible plastic tube (Fig. 1). The connection of the pressure biofeedback unit to digital pressure transducers has been reported in previous studies [14, 20, 30].

Then, the LCD screen of the digital pressure gauge was turned to the subject, who was instructed to gently and slowly perform a nodding action to elevate the target pressure from 20 to 22 mmHg and to hold for 3 s before relaxing and returning to the initial position. During the movement, the examiner requested that the subjects slowly feel the back of their heads to slide on the bed. This process was repeated through each stage of 2 mmHg increments of the test until 30 mmHg.

During the test, the examiner provided visual and verbal cues to guide the process with a correct technique and palpated the muscle activity of the superficial cervical flexors (i.e., sternocleidomastoid and scalene muscles) to avoid their use. Other signs of compensation or poor activation, including neck retraction or a lack of progressive increment of the craniocervical flexion ROM necessary for the CCFT, were monitored by an independent assessor. Neck retraction was prevented based on the procedure described by Falla et al. [30]. The head of the patient rested on a pressure sensor consisting of a thin pressure sensitive textile mat (Sensing Tex^®^, Pressure mat dev kit, Barcelona, Spain), which displayed the pressure values in real-time on a computer screen. Any increase in pressure greater than 0.75 kg was considered neck retraction compensation and implied repetition of the test or exclusion of the subject due to unsuccessful performance of the five stages of the CCFT. This method for measuring neck retraction as a compensation during the CCFT (i.e., increments in weight of 0.75 kg) has been described in previous research [30].

During all of this procedure, a small (4 cm × 4 cm × 8 cm), light (< 200 g) wireless inertial sensor [35] (Werium Solutions©, Madrid, Spain) was adhered to the centre of the forehead of the subject, defined as the place where the lines that bisect the forehead longitudinally and horizontally cross. This sensor provided real-time monitoring of the progressive increment of the craniocervical flexion ROM necessary for the CCFT, which was displayed on a computer. This inertial sensor technology has previously shown good excellent intra-rater and inter-rater reliability in the measurement of cervical ROM [35, 36].

Secondarily, the sensor system automatically recorded the cervical ROM achieved in each stage of the CCFT, represented as the degrees of motion of flexion from the starting neutral position of the neck, when the sensor was calibrated at 0.

The same independent assessor who monitored the pressure of the head against the pressure mat also monitored and recorded cervical ROM. Therefore, this assessor handled two computers connected by Bluetooth in real-time to the pressure and inertial sensors in order to detect whether any retraction compensation or insufficient craniocervical flexion occurred. Subjects who had any evidence of an incorrect execution of the CCFT during the initial screening procedure (i.e., warm up) were excluded from the study.

Figure 1 shows the placement of the pressure biofeedback unit, the inertial sensor and the pressure textile mat used in the measurement process.

### Testing procedure

The process of performing the CCFT described above was used at the screening stage to select the subjects for participation and also to evaluate the reliability of the craniocervical flexion ROM measurement associated with each stage of the CCFT. Examiners A or B randomly performed the CCFT at the initial selection stage. Then, the subjects included in the study attended the first session in which both examiners A and B performed the CCFT test once for each subject in a randomized order (Research Randomizer: https://www.randomizer.org/). Both examiners were mutually blinded to the results of the other examiner. This procedure was subsequently repeated by examiners A and B in a second session with an interval of 1 week, allowing for the calculation of the intra-rater and inter-rater reliability. Examiner C calibrated the inertial sensors and monitored and recorded the cervical ROM in all tests.

Immediately before the performance of the CCFT, examiners A and B also requested that subjects perform three full range active craniocervical flexion actions, which were also recorded by the inertial sensor. The measurement of the full ROM before the CCFT has been previously investigated using digital imaging techniques [30]. It allows for calculations of the percentages of the full ROM necessary to achieve each stage of the CCFT.

**Fig. 2:**
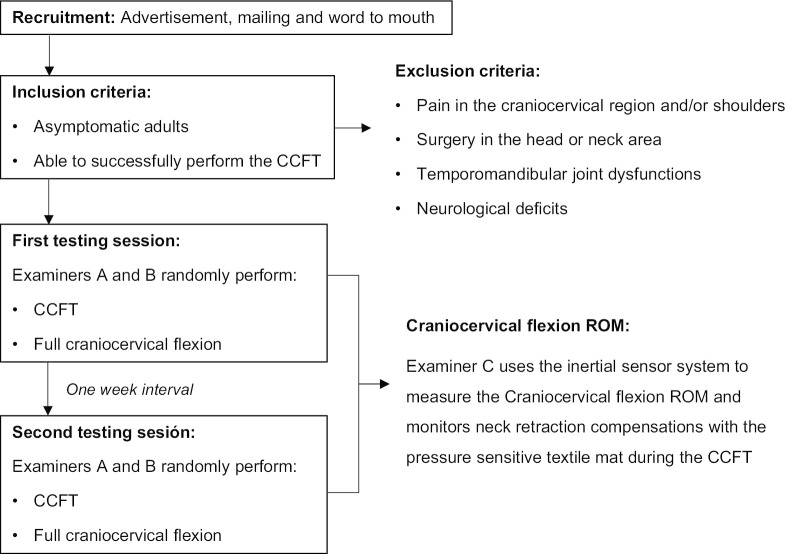
Flowchart of the study protocol

A flowchart of the whole testing protocol is shown in Fig. 2.

### Data analysis plan

All data were analysed using the Statistical Package for Social Sciences software version 24.0 (SPSS Inc, 233 S Wacker Dr, 11th Fl, Chicago, IL 60606, USA). The normal distribution of quantitative data was assessed by the Kolmogorov–Smirnov test, and the mean and standard deviation (SD) for each normally distributed quantitative variable were analysed. The data corresponding to the ROM necessary to achieve each stage of the CCFT was expressed in degrees as means, SDs, 95% confidence intervals (CIs) and as a percentage of the full range of the craniocervical flexion ROM. The reliability of the craniocervical flexion ROM measurement associated with each of the five stages of the CCFT was calculated with intraclass correlation coefficients (ICCs) based on a two-way random effects model, a single rater type and an absolute agreement definition [37]. It included the analysis of “intra-rater” reliability (trial 1 versus trial 2) and “inter-rater” reliability (examiner A versus examiner B) of the measures. The standard error of measurement (SEM) was calculated considering the ICC, using the formula SEM = SD × $\sqrt{1-\mathrm{ICC}}$ [38]. Differences between the stages of the CCFT in the amount of craniocervical flexion ROM were analysed by one way repeated measures analysis of variance (ANOVA).

### Sample size

International guidelines for reproducibility and validity research have reported that 40 subjects can be sufficient for reproducibility studies [33]. In our study, the sample size was calculated considering two raters, an expected ICC of 0.9 with 95% confidence interval (CI), and a confidence level of 0.05. Based on this calculation, 56 subjects were finally estimated to be included [39].

## Results

Seventy-five participants were screened for possible eligibility. Nineteen of them were excluded based on the fulfilment of any of the following criteria: history of pain in the craniocervical region and/or shoulders at the time of the measurement or during the previous year (n = 9), history of surgery in the head or neck area (n = 1), previous diagnosis of TMDs or headaches (n = 3) or not being able to successfully perform the five stages of the CCFT (n = 6). Fifty-six participants (18 males, 23 females; mean [SD] age, 21.8 [3.45] years) were included in the study and successfully completed the study protocol.

The data corresponding to the ROM necessary to achieve each stage of the CCFT and the percentage of the full craniocervical flexion ROM is detailed in Table 1. The analysis of “intra-rater” reliability (trial 1 versus trial 2) and “inter-rater” reliability (examiner A versus examiner B) of the measures is shown in Table 2.

**Table 1:** Descriptive data of craniocervical flexion ROM measures in each stage of the CCFT

Stage of the CCFT	1st measure		2nd measure	
	ROM: Mean ± SD [CI] in degrees	% of full ROM (Mean ± SD)	ROM: Mean ± SD [CI] in degrees	% of full ROM (Mean ± SD)
Examiner A				
22 mmHg	3.2 ± 1.3 [2.8–3.6]	26.8 ± 13.3	3.2 ± 1.4[2.8–3.7]	24.0 ± 9.8
24 mmHg	4,6 ± 1.5 [4.1–5]	37.5 ± 15,5	4.6 ± 1.8 [4.1–5.2]	34.0 ± 11.3
26 mmHg	5.7 ± 1.9 [5–6.3]	47.4 ± 17.7	5.9 ± 2.0 [5.3–6.5]	42.2 ± 11.9
28 mmHg	6.6 ± 2.1 [5.9–7.3]	54.9 ± 19.2	6.9 ± 2.2 [6.2–7.6]	49.9 ± 13.7
30 mmHg	7.6 ± 2.2 [6.9–8.3]	62.7 ± 20.8	7.8 ± 2.4 [7.1–8.6]	57.7 ± 17.2
Full ROM	13.4 ± 4.2 [12.1–14.8]	–	14.5 ± 4.1 [13.2–15.8]	–
Examiner B				
22 mmHg	3.2 ± 1.5 [2.8–3.7]	27.0 ± 13.0	3 ± 1.5 [2.5–3.4]	23.9 ± 11.2
24 mmHg	4.7 ± 1.9 [4.1–5.2]	37.6 ± 18.5	4.4 ± 1.7 [3.9–5]	33.8 ± 13.7
26 mmHg	5.8 ± 2 [5.2–6.5]	46.6 ± 20.1	5.5 ± 1.9 [4.9–6.1]	41.7 ± 13.8
28 mmHg	6,8 ± 2.3 [6.1–7.6]	53.9 ± 23.0	6.5 ± 2.1 [5.8–7.1]	49.4 ± 16
30 mmHg	7.7 ± 2.4 [6.9–8.4]	60.8 ± 24.1	7.4 ± 2.3 [6.7–8.1]	55.5 ± 16.7
Full ROM	13.9 ± 4.5 [12.5–13.4]	–	14.6 ± 4.2 [13.3–16]	–

mmHg millimetres of mercury

**Table 2:** Reliability of craniocervical flexion ROM measures in each stage of the CCFT

	Examiner A		Examiner B	
	ICC (95% CI)	SEM	ICC (95% CI)	SEM
Intra-rater reliability				
22 mmHg	0.85 (0.71–0.92)	0.94	0.87 (0.79–0.94)	1.00
24 mmHg	0.81 (0.73–0.89)	1.28	0.83 (0.78–0.94)	1.39
26 mmHg	0.90 (0.81–0.95)	1.19	0.88 (0.79–0.94)	1.28
28 mmHg	0.95 (0.90–0.97)	0.94	0.90 (0.81–0.96)	1.32
30 mmHg	0.92 (0.84–0.96)	0.91	0.90 (81–0.95)	1.41
Full ROM	0.83 (0.74–0.92)	3.21	0.86 (0.73–0.92)	3.06

	1st measure		2nd measure	
	ICC (95% CI)	SEM	ICC (95% CI)	SEM
Inter-rater reliability				
22 mmHg	0.80 (0.63–0.89)	1.12º	0.78 (0.67–0.86)	1.13º
24 mmHg	0.84 (0.72–0.92)	1.19º	0.83 (0.69–0.91)	1.32º
26 mmHg	0.90 (0.81–0.95)	1.17º	0.88 (0.77–0.93)	1.28º
28 mmHg	0.91 (0.83–0.95)	1.26º	0.90 (0.81–0.94)	1.30º
30 mmHg	0.92 (0.84–0.96)	1.24º	0.90 (0.82–95)	1.39º
Full ROM	0.86 (0.73–0.92)	2.29º	0.96 (0.92–0.98)	1.62º

mmHg millimetres of mercury

**Fig. 3:**
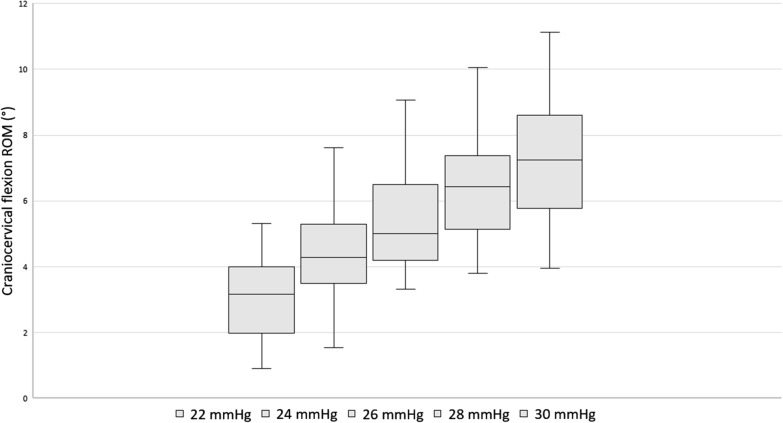
Box and whisker plot showing the 25th, 50th and 75th percentile and upper and lower values of craniocervical flexion ROM in each stage of the CCFT (extracted from data which showed higher reliability from the two examiners)

All ICC values indicated good or excellent reliability of the measures (Table 2). The descriptive data on the ROM and percentage of the full range showed a progressive increase of the amount of craniocervical flexion ROM necessary to achieve each stage of the CCFT (Table 1 and Fig. 3). Figure 1 shows a representation of the mean ROM and mean percentage of the full range (only including the data from Table 1 which showed higher reliability from the two measures of both examiners).

The ANOVA showed a significant effect for time (P < 0.001), representing a progressive increase of craniocervical flexion ROM associated with each successive stage of the CCFT. Post hoc analysis showed that differences between stages were also statistically significant (P < 0.001). The ROM necessary to achieve each stage of the CCFT is detailed in Table 1.

The data on the ROM and percentage of the full range necessary to achieve each stage of the CCFT showed a large variability between participants, evidenced by the large SD and the wide CI values of the measures (Table 1). Although the reliability of the measures was good or excellent, the large SD has also yielded SEM values that are too high considering the small differences of ROM mean values in between consecutive stages (Table 1).

## Discussion

### Inertial sensors to objectively monitor the ROM associated with the CCFT

The use of technology is becoming an important method to evaluate cervical sensorimotor control [40]. This is the first study to investigate a system of inertial sensors to measure the ROM associated with the CCFT. However, the variability of the craniocervical flexion ROM was high and thus limits the possibility to accurately standardize a target ROM associated with each stage of the CCFT.

Our results showed that wearable inertial sensors were a reliable method to measure the craniocervical flexion ROM associated with each stage of the CCFT. One of the three components of the performance of the CCFT using the pressure biofeedback unit is to visually assess and confirm that the rotation of the head in the sagittal plane proportionally increase with the progressive stages of the test [15, 24]. However, the ability of the examiner to accurately observe the anterior rotation of the head is subjective, but the use of inertial sensors provides a precise electronic data record and feedback both for patients and examiners. Therefore, their use as described in this study could be implemented in future research and clinical practice as a user-friendly option to objectively monitor the ROM associated with each stage the CCFT, while computer feedback could provide performance-guidance for the examiner.

The execution of the CCFT requires that the clinician observes whether an altered movement strategy of a subtle retraction rather than a pure anterior sagittal rotation, occurs. This altered movement strategy has been associated with an average 5% reduction of the craniocervical flexion ROM at each stage of the CCFT [15]. The detection of this aberrant strategy is challenging for clinicians and it could also be facilitated by the use of inertial sensors, since they detect an absence of craniocervical flexion even though when there is an increase of the target pressure. This would indicate that a retraction movement is occurring without any craniocervical flexion.

Similar to previous research using digital imaging [14, 15, 30, 31], the present study has reliably observed that the amount of craniocervical flexion progressively increases during the five successive stages of the CCFT. Full craniocervical flexion ROM values observed in our study (14.6° ± 4.2°) are in line with those observed by Uthaikhup and Jull in a young healthy population (14.3º ± 5.2º) [31]. However, they seem significantly higher than those previously reported by Falla et al. [30] in young healthy adults (8.5º). This difference could be explained by the instructions given to the patients during the performance of the full range of active craniocervical flexion. With regard to the ROM values reached in each stage of the CCFT, the percentage of full craniocervical flexion observed in our study (22 mmHg: 27%; 24 mmHg: 37.5%; 26 mmHg: 47.4%; 28 mmHg: 54.9%; and 30 mmHg: 62.7%) also seem quite similar compared with those previously observed by Uthaikhup and Jull [31] in young healthy individuals. However, they seem slightly lower when compared to Falla et al. results [30], especially for the last two stages (22 mmHg: 24.9%; 24 mmHg: 41.9%; 26 mmHg: 55.8%; 28 mmHg: 66.5%; and 30 mmHg: 76.6%). These differences may be explained by the fact that participants in our study had increased values of the full range of the craniocervical flexion ROM, and therefore, the percentage of the full ROM necessary to reach the target pressure level was reduced.

### Potential use of inertial wearable sensors as an alternative to test and retrain the DCF muscles

To the authors’ knowledge, no previous research has investigated inertial sensors to measure the ROM associated with the CCFT or as an option to test and retrain the DCF muscles. The CCFT is limited for the patient in a supine position, since the air-filled pressure sensor needs to be compressed between the cervical lordosis and the bed. Although training approaches of the DCF muscles based on the CCFT have been demonstrated to improve the muscle function, previous research has observed that craniocervical flexor training in the supine position using feedback from an air-filled pressure sensor did not produce changes in muscle activation during a standing functional upper limb task. Thus, it was suggested that rehabilitation of the cervical muscles should be extended to include training in functional postures and tasks [32].

The results of the current study have shown that the variability of craniocervical ROM associated with each stage of the CCFT observed between subjects limits the possibility to standardize a set of targets of craniocervical flexion ROM equivalent to each of the pressure targets of the pressure biofeedback unit. This variability might be explained by various factors, such as anthropometric characteristics, cervical laxity and anatomical characteristics of the cervical lordosis or the occiput prominence, considering that the stages of the CCFT specifically monitor the slight flattening of the cervical lordosis that compresses the pressure biofeedback unit sensor.

The inclusion of young healthy participants in the present study may have reduced this variability, so larger variability ranges would be expected in patients who cannot successfully complete all stages of the CCFT or with pain conditions associated with poor sensorimotor control, losses of segmental mobility or alterations in central processing. Larger variability ranges of craniocervical flexion ROM during the CCFT have also been reported in the elderly and considered indicative of impaired fine motor and cognitive skills [31]. Moreover, differences between each of the stages of the CCFT \ in terms of the mean data of craniocervical flexion ROM and percentage of full ROM are always small (< 1.5º or 11% of the difference between two consecutive stages). Therefore, this factor also limits the possibility to accurately standardize a set of targets of ROM equivalent to each of the pressure targets of the pressure biofeedback unit, since the SD of the measures and the SEM of each stage tend to overlap.

Further research is needed to analyse the relationship between different targets of craniocervical ROM and the amplitude of DCF muscle activity, allowing for the standardization of a set of targets of craniocervical ROM associated with specific amplitudes of DCF muscle activity. Therefore, future research could investigate whether inertial sensors associated with computer feedback could provide an alternative option to test and retrain the DCF muscles in multiple positions or functional postures and tasks, accomplishing the needs of clinicians who treat patients with multiple craniocervical pain conditions.

### Limitations of the study

This is the first study to investigate a novel inertial technology that could assist clinicians when performing the CCFT in patients with neck pain and associated conditions (i.e., headache, TMDs and craniofacial pain). However, the present study has various limitations. First, the results of this study are limited to the specific characteristics of the study population, which included asymptomatic, young Caucasian participants from a university community. Although this type of sample may be appropriate to describe the normative values of craniocervical flexion ROM that should be achieved in normal conditions during the CCFT, future research could investigate whether its performance is influenced by age, anthropometric features or anatomical characteristics of the cervical spine and the cranium. Previous research has observed that healthy elders showed larger variability in craniocervical flexion ROM for the five stages of the test as compared to young subjects [31]. Second, the muscle activation of superficial muscles while performing the CCFT was not monitored by electromyography. This could have allowed for an objective measure of subjects who could have been excluded from the study due to superficial muscle activity. Third, some factors could have provoked a different performance of the CCFT in each trial separated by 1 week, such as the previous training on the test or the physical and psychological status of the patient on the day and time they were measured.

### Implications for practice and future research

Inertial sensors are a user-friendly option to objectively monitor the ROM associated with each of the pressure targets of the pressure biofeedback unit during the CCFT, providing a real time computer feedback. Therefore, their use as described in this study could be implemented in clinical practice and research to confirm that the craniocervical flexion ROM proportionally increases with the progressive stages of the CCFT and to detect any aberrant strategy.

Further research is needed to evaluate the specific muscle activity of the DCF muscles associated with various targets of craniocervical ROM to accurately determine which craniocervical flexion ROM best targets the DCF muscles.

## Conclusions

The use of inertial sensors is a reliable method to measure the craniocervical flexion ROM associated with the CCFT. It can allow future research and clinicians to objectively monitor the quality and range of head sagittal plane rotation during the CCFT performed with the pressure biofeedback unit, in order to confirm that it proportionally increases with progressive stages of the test and to identify retraction aberrant strategies.

There is a high variability between subjects on the amount of craniocervical ROM necessary to achieve each stage of the CCFT, which limits the possibility to standardize a set of targets of craniocervical flexion ROM equivalent to each of the pressure targets of the pressure biofeedback unit.

## Data Availability

The datasets generated during the current study are available from the corresponding author upon request.

## Abbreviations

CCFT: Craniocervical flexion test
DCF: Deep cervical flexors
ICC: Intraclass correlation coefficient
ROM: Range of motion
